# Impact Strength of Preplaced Aggregate Concrete Comprising Glass Fibre Mesh and Steel Fibres: Experiments and Modeling

**DOI:** 10.3390/ma15155259

**Published:** 2022-07-29

**Authors:** Nirmal Ponnambalam, Sarathkumar Thangavel, Gunasekaran Murali, Nikolai Ivanovich Vatin

**Affiliations:** 1 Department of Civil Engineering, Government College of Technology, Coimbatore 641013, India; nirmalponnambalam113@gmail.com; 2 Department of Civil Engineering, Government College of Engineering, Dharmapuri 636704, India; tsarath.15@gmail.com; 3 Peter the Great St. Petersburg Polytechnic University, 195251 Saint Petersburg, Russia; vatin@mail.ru; 4 Division of Research & Innovation, Uttaranchal University, Dehradun 248007, India

**Keywords:** impact energy, fibre, glass fibre mesh, failure, modelling, concrete

## Abstract

Concrete is the most widely used and most affordable construction material. The structural damage that concrete cracks and fractures may cause can be severe. These concerns have lately been alleviated by new developments in fibre concretes. Recent advancements in fibrous concrete and its evolution have been rapidly drawing researchers’ attentions worldwide, which motivates the development of a new type of composite with superior impact resistance. Preplaced aggregate fibrous concrete (PAFC) is a revolutionary composite comprising a higher dosage of fibres. It has outstanding impact resistance that surpasses those of traditional fibrous concrete. The impact behaviour of PAFC in addition to glass fibre mesh (GFM) has not been investigated thoroughly. To fill this research gap, this study investigates the impact performance of three-layered PAFC comprising steel fibres and GFM insertion. Eight different mixtures were prepared and can be divided into two groups. In the first group, specimens were made with 4% fibres and two single, double and triple layers of GFM insertion between the three-layered concrete. The second group of specimens was reinforced with 5, 2 and 5% steel fibres at the top, middle and bottom layers, respectively. However, the GFM insertion scheme for the second group was the same as the first. Rectangular specimens of size 500 × 100 × 100 mm were cast and tested against drop weight impact. The parameters studied were cracking impact numbers, failure impact number, ductility index and failure patterns. In addition, an analytical model was used to evaluate the impact failure energies. Results indicate that the combined action of steel fibre and GFM exhibited an excellent impact resistance. Increasing the number of GFM insertions between the specimen layer led to increased impact strength. The dose of the fibres utilized in the outer layer of the PAFC was increased, resulting in the material having a higher impact resistance. The cracking impact numbers improved from 28 to 40%, and failure impact numbers ranged from 58.8 to 92.2% when the GFM insertion numbers increased from one to three.

## 1. Introduction

Concrete is often considered the most essential building material in the world [1]. Recent years have witnessed an increase in the use of concrete or fibre concrete in military structures and essential infrastructure to withstand external assaults [2]. Concrete may be subjected to various impact loads over its life cycle [3]. Engineers deal with a wide variety of impact situations. Some examples include rocks falling from above, ships and barges striking bridge piers, industrial flooring being impacted by moving machinery or vehicles, and equipment crashing to the ground [4,5]. As a result, the frequency and probability of these effects become significantly more substantial. Hence, evaluating concrete’s impact load performance is critical for creating a secure and cost-effective structure [6,7]. A lack of tensile strength, flexibility, and impact energy mean that non-fibrous concrete cannot resist successive impacts [8]. As a consequence, the incorporation of fibre into concrete produces a much greater level of ductility [9], impact resistance [10] and toughness [11]. It also mainly prevents crack extension and improves post-crack resistance without leading to fracture.

Prepacked aggregate composite (PAC) was invented due to the advancement of fibrous concrete and has since grown in popularity [12,13]. Due to the unique production method, preplaced aggregate fibrous concrete (PAFC) is a different form of composite that varies from ordinary fibrous concrete [14]. The first process involves coarse aggregates being combined with fibres, placed in a framework, then injected with grout to fill gaps between the particles. A little quantity of grout is all that is needed to be fully mixed and pumped during this operation, decreasing the amount of time and effort necessary. The technique of PAFC makes it possible to build thick monoliths without the need for consolidation procedures, vibration, or compaction, which results in time, financial, and resource savings. PAFC is quickly becoming the material of choice for a wide range of applications as a result of its sustainability benefits and cost-effectiveness it offers. Radiation shielding in nuclear structures, undersea structures, tunnels, and mass concrete constructions are all examples of applications [15]. Several researchers have thoroughly investigated the impact resistance of PAFC. Prasad and Murali [16] state that there are increases of 844% and 2700% in the number of impacts generating cracking and failure in steel fibre-based PAC specimens, respectively, compared with control specimens. Mohammadhossein et al. [16] used waste polypropylene carpet fibres to create a unique PAFC and test its strength and transport properties. Some of the cement was substituted with palm oil ash. The gravity approach made six combinations of 30 mm-long fibres ranging in percentage from 0% to 1.25%. The same fibre dosages were used to produce six more batches, and the grout was pumped into the spaces between the aggregates. Their results show that carpet fibre-reinforced specimens have a lower compressive strength than those without fibres. When using 0.75% fibre, their tensile strength in the gravity and pumping methods is greatly enhanced. The tensile strength of fibre doses greater than 0.75% was reduced by the loss of fluidity caused by a lower degree of compaction. Fibrous concrete has evolved from PAFC to multi-layered concrete throughout time with other combinations such as GFM. 

An ecologically friendly PAFC has been suggested and tested by Ramkumar et al. [17]. The specimens used for the impact testing included three layers, each of which had fibre doses of 6, 3 and 6% applied, respectively, to the top, middle and bottom layers. Crimped fibres were used to strengthen the central layer and hooked end fibres were used to reinforce the outer ones. According to the test findings, the layered PAFC was much more impact resistant than the single-layer PAFC. Slab impact behaviour was studied by Murali and Ramprasad [18] for PAFC slabs in the case of a mass impact. The three-layered slab’s top, middle, and bottom layers were reinforced with 4%, 2%, and 4% fibre dosages, respectively. The findings show that multilayer PAC has a greater impact energy absorption capacity, eliminates its brittleness, and delays the emergence and development of cracks. 

Glass and basalt fibre reinforcing polymer meshes were compared by Diab et al. [19] in their abilities to control alkali–silica reaction expansion and improve the mechanical characteristics of concrete affected by alkali–silica reaction. According to the findings, an increase in compressive strength was seen in the range of 4.6 to 39% for basalt fibre mesh and from 6.5 to 50% for glass fibre mesh. According to research by Baraghith et al. [20] the increases in shear capacity and ductility of reinforced RC beams made with intermittent glass fibre textile mesh layers and strain-hardening cementitious composite strips were in the range of 47 to 142% and 21 to172%, respectively. The cracking load was postponed, and the ultimate shear capacity and the ductility index rose when the number of glass fibre textile mesh layers contained inside strain-hardening cementitious composite strips increased. Man et al. [21] reported that the crack resistance of self-stressing concrete was improved by reinforcing with glass fibre composite mesh. According to the findings of Liao et al. [21], woven glass fibre mesh can potentially boost the energy absorption of polyurea coating by 2.5 times and reduce the damage sustained by a retrofitted reinforced concrete slab, particularly if the slab collapses and suffers extensive damage.

Murali et al. [22] reported that the crack arresting capability was shown to be improved and impact energy increased with an increase in the diameter of the GFM between the expanded clay aggregate concrete layers. The GFM diameter was raised from 50 to 150 mm, which resulted in a rise of 49 to 60% in the cracking impact number and 117 to 152% in the failure impact number. Rithanyaa et al. [23] researched the impact resistance of three-layer PAFC slabs to the impact of drop weight. Three percent of steel fibre was employed in the layers on the outside, and 1.5% in the middle. In addition, GFM mesh was inserted between the layers. The investigation found that the joint action of the glass fibre and steel fibres halted the formation of cracks, exhibiting superiority in the absorption of impact energy and slowing the formation of cracks. Abirami et al. [24] investigated the effect that GFM has on three-layered PAFC when it is subjected to the impact of drop weight. GFM, with varying diameters ranging from 50 to 150 mm, was introduced from one to four numbers at the top layer and from four to one at the bottom layers, and inversely. A hooked-end steel fibre dose of 2.5% was used to produce the PAFC. As a consequence of increasing the diameter of the GFM in PAFC, impact energy increased by a greater percentage. When comparing the TSFC specimens with 50, 75, 100, and 125 mm diameter glass fibre mesh, the specimen with 150 mm diameter glass fibre mesh showed the highest impact energy by approximately 43.5, 34.3, 18.3, and 7.2%, respectively. Pan et al. [25] investigated the addition of GFM in engineering cementitious composite and evaluated the fatigue performance when applied in runways. According to their findings, GFPM-reinforced ECC can endure 800 times more hits before cracking than ordinary concrete pavement and can survive 30,000 hits before suffering damage. 

Several studies have been conducted to evaluate concrete impact resistance using different types of fibres. Most of these studies were focused on evaluating the impact strength using small cylindrical specimens and limited focus on beams under bending. Moreover, GFM insertion in concrete slabs and panels was studied to determine their load carrying capacity. The effect of GFM insertion in concrete under impact loading is minimal. The combined effect of steel fibre and GFM insertion in PAFC under impact loading is unexplored and needs special attention. 

## 2. Research Significance

There is a paucity of understanding concerning the impact performance of PAFC that comprises steel fibres in addition to GFM in-between their layers of specimens. As a result of this knowledge gap, academics in this subject have shifted their attention to this current hot topic of study. Unfortunately, the available literature in this area is rather sparse, necessitating further attention. In terms of bending impact, the impact performance of layered PAFC is currently unknown and requires more investigation. Consequently, this work aimed to gain full knowledge of the three-layered PAFC, including GFM between the layers and steel fibres. The three schemes of GFM insertion (layers 1, 2 and 3) are introduced between the PAFC layers. Moreover, the outer layers of PAFC contain higher fibre content and the inner layers contain medium fibre content to facilitating higher impact resistance than unform fibre content throughout the cross-section. This three-layer modification in PAFC, together with steel fibre and GFM addition, is the work’s novelty. A new composite material, the layered PAFC, is described as having a strong crack inhibiting capability when subjected to impact loads.

## 3. Experimental Methods

The steps followed in conducting the experimental studies are as follows; first, the three-layered PAFC concrete beams were prepared with the grout injection technique. A step-by-step casting procedure was adopted to complete each layer of PAFC comprising steel fibres. After completing the first layer of PAFC, a number of GFMs are placed above the first layer, followed by the casting of the second layer. Another number of GFMs are placed above the second layer, followed by the completion of the third layer. All beams were subjected to the drop weight impact test to measure the cracking and failure impact numbers, and energies were calculated. In addition, analytical modelling was used to predict the failure impact energies and compared with the experimental findings.

### 3.1. Raw Materials

**Figure 1:**
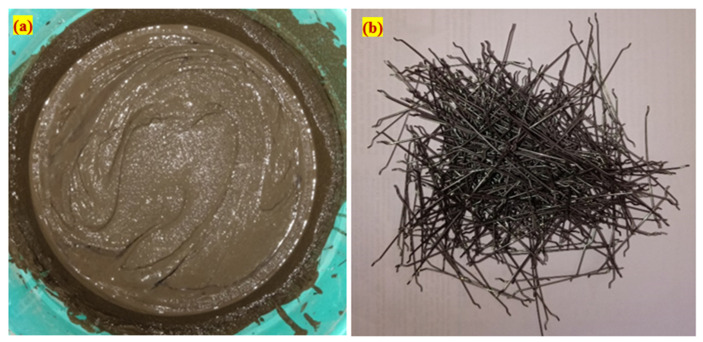
Appearance of fibre and prepared grout. (**a**) Cement grout and (**b**) steel fibres.

**Figure 2:**
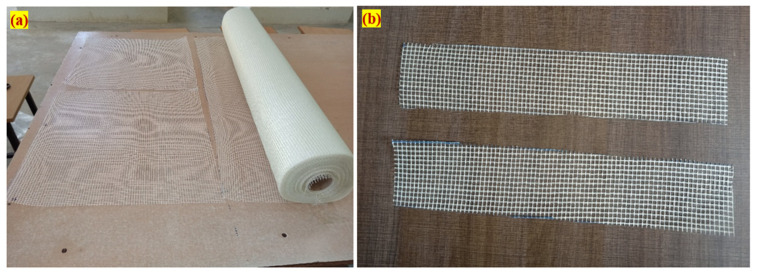
Details of glass fibre mesh. (**a**) Mesh roll and (**b**) rectangular pattern of mesh.

- An Ordinary Portland Cement (OPC) was utilized in this study, satisfying the IS: 12269–1987 [26]. The specific surface area of the cement utilized was 318 kg/m^2^ and its specific gravity was 3.14.
- The fine aggregate was sourced from a local natural river with a specific gravity of 2.65 and a fineness modulus of 2.41 in accordance with IS: 383–2016 [27]. An ASTM C939 [28] compliant grout with fine aggregate particles less than 2.36 mm was used. Thus, the superior gravity flow could accomplish a great flow through the skeletal aggregate. The grout was prepared with good flowability, as shown in Figure 1a.
- The dimension of the granite gravel that was employed for the coarse aggregate was 12.5 mm in size. The coarse aggregate had a bulk density of 1700 kg/m^3^, water absorption value of 0.56% and specific gravity of 2.69.
- In order to improve the flowability of the grout and satisfy the criteria for the efflux time, a superplasticizer called Tec Mix 640 sourced from Techny Chemy, Trichirapalli, India was used. The two doses of chosen superplasticizers were 0.3 and 0.6 percent (by cement weight), with the former being used on non-fibrous specimens and the latter being used on fibrous specimens.
- Hooked end fibre, 30 mm long and 0.5 mm in diameter, was used with a tensile strength of 1400 MPa and Youngs modulus of 210 GPa. The fibre used in this research is shown in Figure 1b.
- Grids of two-way glass fibre reinforcement spaced at 5 mm intervals and weighing 125 g/m^2^ per unit area were used in this study. The GFM had a density of 2.58 g/cm^3^, tensile strength of 3.445 GPa, Youngs modulus of 72.3 GPa, elongation of 4.8% and poison’s ratio of 0.2. The GFM roll was sourced from Virendera Textiles, Uttar Pradesh, India, as shown in Figure 2a, and was cut for a rectangular shape, as shown in Figure 2b.

### 3.2. Mixing Combinations

In this experiment, eight identical mixtures were made, each with the same water-to-cement (w/c) and cement-to-sand (c/s) proportions. In order to satisfy the 35–40 2 s efflux time [28], a significant number of trial grout mixes were prepared to optimize the water-to-cement and cement-to-sand ratios, as well as the quantity of superplasticizer necessary. The first mixture was F0, which indicates the non-fibrous reference specimens. The second mixture was designated as F-M0, which indicates the fibrous specimens without GFM insertion. The mixtures 3–5 were designated as F-M1, F-M2 and F-M3, respectively, and a fibre dosage of 4% was used to the full depth of the beam. M1, M2 and M3 indicate the number of GFM layer insertions in PAFC. Finally, the last three mixtures were designated as LF-M1, LF-M2 and LF-M3. The fibres used for the top, middle and bottom layer of PAFC were 5, 2 and 5% (by volume), respectively. The GFM pattern is the same as for mixtures 3–5. The detailed mixing combination employed in this study is demonstrated in Table 1.

### 3.3. Specimen Preparation

**Figure 3:**
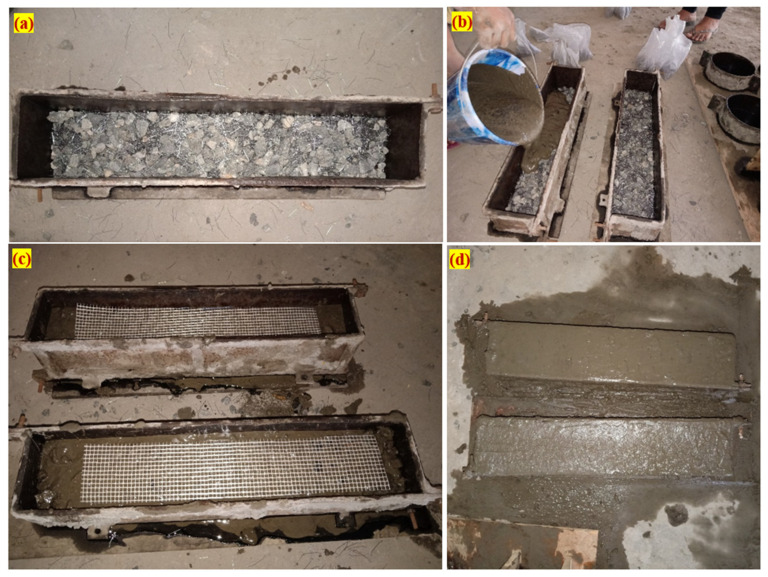
Casting process. (**a**) Mild filled with fibres and aggregates, (**b**) grout injection, (**c**) GFM addition, and (**d**) beam after finishing.

**Figure 4:**
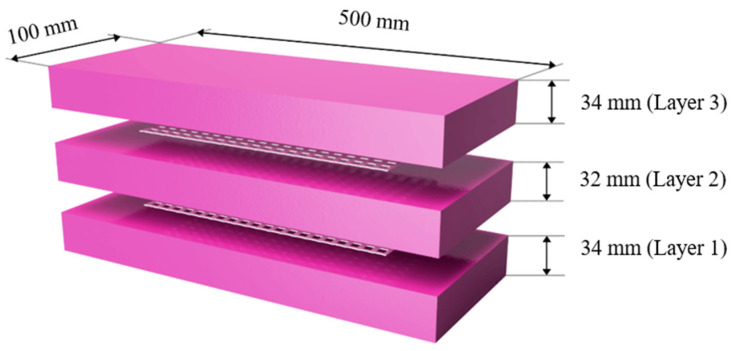
GFM insertion location.

The impact strength of PAFC was evaluated by fabricating a beam specimen. A rectangular specimen of length 500 mm and cross-section of 100 mm × 100 mm was made to test the PAFC’s impact resistance. The formwork must be carefully cleaned and oil-coated on all inside surfaces before the fibres and aggregates fill the mould. The frameworks were then stuffed with coarse gravel and fibres, creating natural skeletons of aggregates (Figure 3a). In the end, a cement grout with a high flowability was poured on top of the natural skeletons, filling the gaps (Figure 3b). The gravity method was used for grouting to fill the voids between the aggregates and the fibres; strong compaction or vibration was not required at any stage of the operation. To avoid honeycombing, a specimen was subjected to light compaction using the 6 mm diameter steel rod. The GFM was inserted after finishing the first layer of PAFC (Figure 3c). It is essential to highlight how the GFM covers 64% of the total area to avoid shearing failure. Subsequently, the casting process is repeated to complete the specimens. The appearance of the fabricated specimen after finishing is shown in Figure 3d. The GFM insertion location in PAFC is shown in Figure 4.

### 3.4. Test Setup

**Figure 5:**
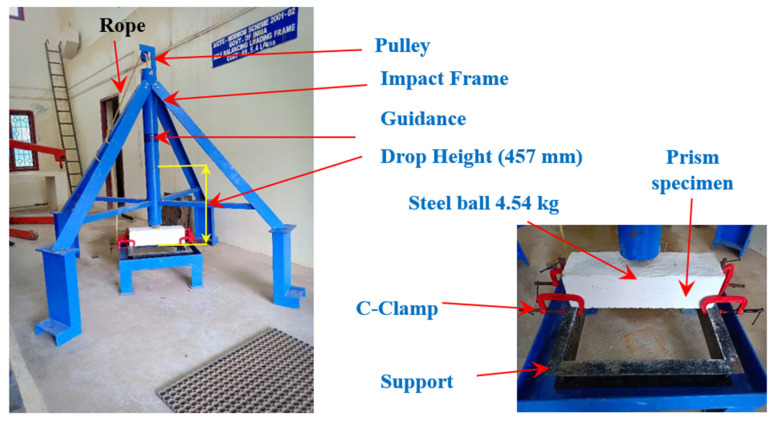
Testing setup for impact strength.

Concrete strength was assessed using a drop-weight impact test recommended by ACI Committee 544 [29]. Tests were simple and consisted of a 4.54 kg steel ball raised vertically from the target specimen’s top surface at 457 mm (Figure 5). The gravity drop test involved repeatedly dropping the ball on a target. Impact tests with drop weights are too simplistic since they do not need any load history, deformations, or vibrations. The first crack (A1) and failure (A2) were determined only by counting the number of impacts. Cracks that start at the specimen’s bottom surface and progress to the top surface are an indication of the failure. Cracking and failure were visible in the specimens when they were visually inspected. This testing method has been used by several researchers to evaluate the impact strength of beam specimens [30,31]. Impact energy is consistently delivered in the same amount with each impact. The impact energies per impact blow are calculated using Equation (1).

(1) Impact energy (U−A)=n×m×g×h

where, *h* is the vertical distance the ball fell, *g* is the acceleration of gravity (9.81 m/s^2^), *m* is the steel ball weight (4.45 kg) and *n* is the number of impacts to the specimen.

## 4. Results and Discussions

The compressive strength was assessed using a 100 mm cubical specimen. The observed compressive strengths of non-fibrous and fibrous specimens were 33.65 and 54.52%, respectively. The influence of GFM on the compressive strength of PAFC were not investigated in this study. 

### 4.1. Impact Strength

**Figure 6:**
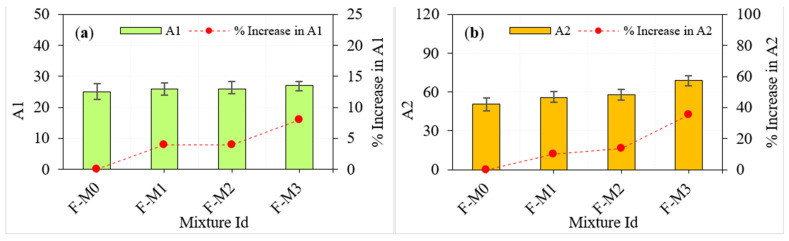
Impact test results of fibrous specimens. (**a**) A1 (**b**) A2.

**Figure 7:**
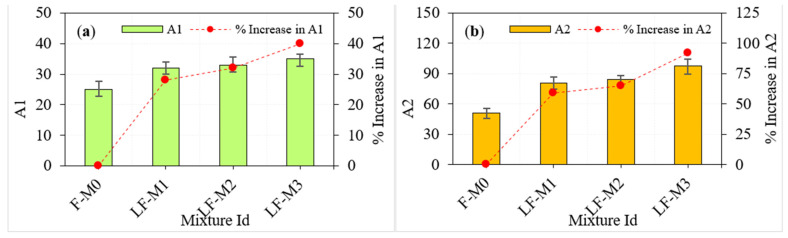
Impact test results of layered fibrous specimens. (**a**) A1 (**b**) A2.

Table 2 provides the findings of the ACI 544 [29] repeated impact test for the plain and fibrous mixes, while Figure 6 and Figure 7 exhibit the results and percentage improvements in impact strength, respectively.

Two effects can be investigated in the presented impact results of this study, which are the effect of GFM with constant fibre dosage in three layers of PAFC, and the effect of GFM with varying dosages of steel fibres. The observed coefficient of variation for A1 and A2 were less for all fibrous concrete, which indicates less scattering in the test results. As seen in Figure 6, increasing the number of GFM layers improved the cracking impact resistance of the F-M0 mixes (which had no GFM). When the A1 value of mixture F-M0 (without GFM insertion) was used as a reference record, the percentages of increase in A1 due to the number of GFM insertions between layers (1, 2, and 3) were approximately 4, 4, and 8%, respectively. As the number of GFM layers increased, the failure impact resistance also exhibited a steady improvement, as shown in Figure 6b. The percentage improvements in A2 of the mixtures F-M1, F-M2 and F-M3 compared with F-M0 were approximately 9.8, 13.7 and 35.3%, respectively. It is clear from the above discussions that increasing the numbers of GFM insertions in PAFC led to an enhancement in the impact strength. We may infer from the above that adding steel fibres and GFM to PAFC can slow down composite degradation when subjected to a drop-weight impact. This might be due to the presence of fibre and GFM inside the composites, which prevents the microcracks from becoming larger and slows down the rate at which the damage spreads [32]. Evenly distributed fibres also improved bridging and reinforcing effects and high cohesion between fibre and matrix, transferring stress from the crack tip to the top specimen surface. The alleviation of the stress level concentration inside the PAFC was the cause of the uniform stress transmission that occurred [33]. All of the PAFC specimens fractured in the bottom middle of their span owing to the initiation and cracking continuing towards the specimen’s top surface, which was followed by the specimens trying to break apart into two separate pieces [34]. Micro-fractures began to show in a highly stressed zone (midspan), and then they spread out into many cracks that moved upward towards the top surface of the specimen. As a result, the process of fibre pull-out started sooner in the weaker fibres. Additionally, the presence of GFM acts as a crack arrester, reducing the possibility of cracks throughout the specimen layers [22].

Incorporating more than 1.5% of hooked-end steel fibre can significantly increase the cracking resistance and boost the failure impact resistance. The only influence that steel fibre had on A1 and A2 was determined by comparing the fibrous mixes and the simple mixtures used as a control (F0). It was observed that the percentage development on A1 was roughly 40% and that on A2 was approximately 750% due to the addition of steel fibres. Steel fibres are reinforced components with a short length and a high tensile strength. These minuscule components are dispersed randomly across the matrix in various orientations to create multidirectional discontinuous microstructural reinforcement grids. The steel fibres’ bridging role would prevent a fracture from forming within the matrix due to impact shock waves. As a result, steel fibres activity delay the propagation of newly generated cracks to the surface for many additional impact strikes. Consequently, the documented number of impact strikes leading to the first surface crack increases.

Varying fibre dosage in three layers of PAFC showed positive results in impact strength, as shown in Figure 7. For the mixtures LF-M1, LF-M2 and LF-M3 that include 5, 2, 5% fibres in three layers, the percentage improvements in A1 compared with that of F-M0 were calculated to be approximately 28, 32, and 40%, respectively. On the other hand, the corresponding percentage developments in A2 were approximately 58.8, 64.7 and 92.2%, respectively. This phenomenon is because the crack propagation may be prevented by a GFM, which absorbs the tensile stresses generated by the cracking process and significantly improves impact resistance. However, increasing the GFM numbers in between the PAFC layers led to an increase in A1 and A2 values. The combined action of steel fibre and GFM is remarkable in delaying the crack propagation and extending the failure of the specimens. At the same time, steel fibres play a role in crack bridging and GFM as a barrier during crack propagation. Adding more fibres to the outer layer of PAFC results in higher impact resistance than the uniform fibres dosage in PAFC. 

Furthermore, providing two layers of GFM in concrete significantly improved impact strength. Pan et al. [25] reported that the GFM should be positioned at the bottom of the pavement so that the mesh is under tension when the top surface is subjected to an impact force. When placed on top of the pavement, the GFM reinforcement has a negligible impact. It is preferable to use two layers of GFM reinforcement in the engineering cementitious composite pavement wherever feasible. According to the findings of Zhang et al. [35], using fibre mesh can significantly enhance the impact resistance of concrete pavement slabs. Pavement reinforcement was most effective using an aramid fibre mesh, whereas it was least effective with a basalt fibre mesh. On the other hand, the impact resistance of concrete pavement slabs is not significantly affected by the mesh size.

### 4.2. Impact Ductility

**Figure 8:**
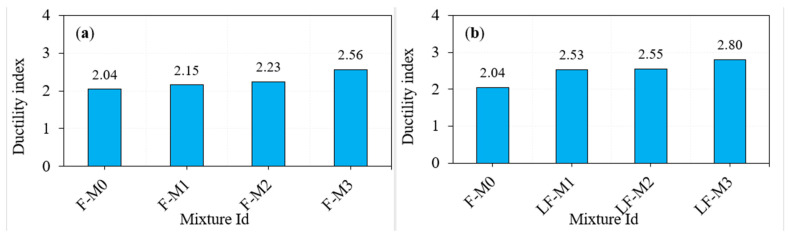
Ductility index. (**a**) Fibrous specimens and (**b**) layered fibrous.

A flexural member’s capacity to absorb plastic energy is defined by ductility, which refers to a specific physical quality. The post-yielding region of the load-displacement curves of members under bending loads is used to compute the plastic region, which begins at the yielding point of the tension reinforcement and ends at failure. This definition was also used to evaluate the influence of fibres and other additives on the post-cracking behaviour of disk specimens exposed to the ACI 544 repeated impact, where various earlier investigations have been conducted [36,37,38]. The ratio of cracking to failure impact numbers (A2/A1) was used to measure impact ductility. Steel fibre and GFM were also used in this investigation to examine the effect on the failure impact resistance of the material. Consequently, the more ductile the combination is, the greater the ratio of A2/A1. The ductility index of the F0 specimen was 1.20 (Table 2), which indicates the specimen behaves in a brittle manner. This brittleness was reduced by using fibres in concrete and the corresponding ductility value for the mixture (F-M0) was 2.04 (Figure 8). Introducing GFM insertion to the PARC resulted in a marginal increase in ductility index. For example, the ductility value for the F-M1, F-M2 and F-M3 mixtures were 2.15, 2.23 and 2.56, respectively. At the same time, changing the fibre dosage in three layers results in marginal influence in increasing ductility index. For the mixtures LF-M1, LF-M2, and LF-M3, the observed ductility values were 2.53, 2.55 and 2.80, respectively. The increased ductility index is due to the better ductility and plastic energy capacity of steel fibres and GFM [39].

### 4.3. Failure Pattern

**Figure 9:**
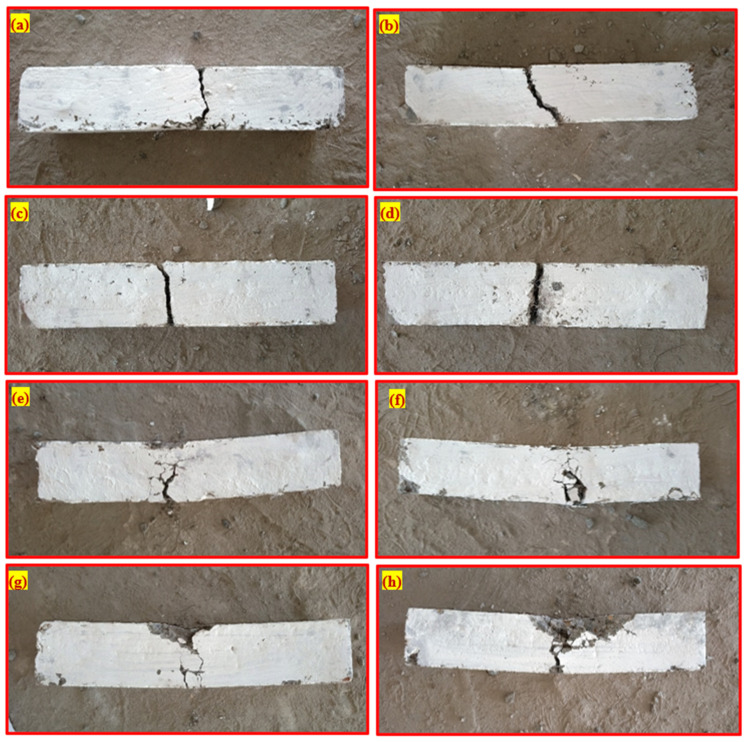
Failure mode of beams. (**a**) F0, (**b**) F-M0, (**c**) F-M1, (**d**) F-M2, (**e**) F-M3, (**f**) LF-M1, (**g**) LF-M2 and (**h**) LF-M3.

Figure 9 depicts the most common failure pattern of the PAFC. After the first crack appeared, the non-fibrous specimen rapidly broke down, suggesting brittle behaviour against impact loads (Figure 9a). This phenomenon arises due to the absence of fibre bridging activity and the brittleness of concrete [40]. At the same time, the F-M0 specimen contains steel fibres and was broken into two pieces, while the impact energy absorption capacity was more. The mixture’s energy absorption capacity was increased when fibres and GFM were added, as seen in Figure 9c–e. The GFM and fibre-based specimens have microfractures that prevented them from collapsing abruptly because of their active nature. The behaviour of these specimens, which was observed, suggested that they could have the ability to keep their structural integrity while also being ductile. The combinations of GFM and steel fibres (higher dosage at outer layers) dramatically altered the failure mechanism of the material, changing it from a single crack to multiple cracks, known as a ductile failure (Figure 9f–h). As a result, these failure patterns demonstrated a specimen’s positive effect when subjected to impact loading. The greater the number of hits that were absorbed, the more likely it was that a fracture zone would develop in the middle of the specimen. Initially, the micro-cracks were formed in the specimens and increasing the number of impacts turned the micro-cracks into multiple micro- and macro-cracks [41,42]. The uniformly distributed fibres also improved bridging and reinforcing effects and high cohesion between fibre and matrix, transferring stress from the crack tip to the top specimen surface [6]. Adding fibres increases the micro- and macro-cracks while also increasing the enormous impact energy [30]. The increasing frequency of collisions led to the production of surface fractures of varying sizes, which occurred due to the enlargement of the fracture zone. As the frequency of impacts increased, these cracks became wider and eventually spread to the top surface of the specimens. The fibre was regularly used to bridge the two different sides of the cracks. However, at the same time, the bonding between the fibre and the cement matrix surrounding it gradually deteriorated, leading to the failure caused by the fibre pull-out [43]. 

### 4.4. Failure Mechanism

**Figure 10:**
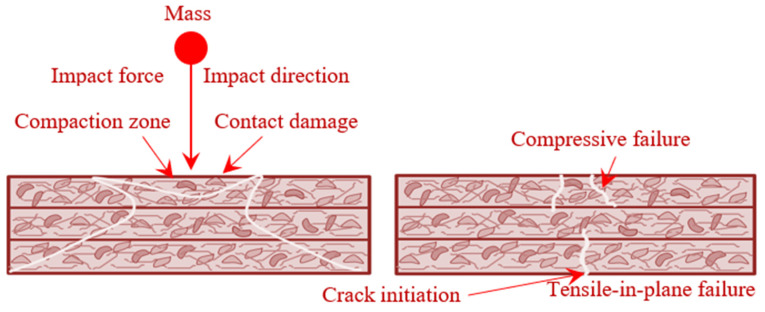
Failure mechanism.

Cracking, shearing, and compaction are the most prevalent causes of concrete fracturing when subjected to impact force [44] (Figure 10). It is possible to anticipate that the concrete will crack in the direction of the forces acting upon it, such as compression, tension, or confining stresses. Nevertheless, the formation of ultimate cracks in the total concrete specimen depends on the concrete’s fundamental qualities, including fibres and GFM. Therefore, even after the initial fracture has appeared, the fibres can still bridge it and distribute the energy to several other locations within the concrete [45]. When the fibres can no longer prevent fractures from forming, they are pulled out, causing damage to the concrete due to the impact stress distribution on concrete [46]. Contact damage, fibre rupturing, matrix failing, and fibre delamination are problems noticed when specimens were subjected to impact testing. This includes the destruction caused by impact applied to specific points on the specimen. Transverse shearing stress/strain causes internal debonding of the material. Failure of the matrix fibres is caused by compressive bending on the impact plane. Finally, tensile bending at the bottom surface causes fibre debonding to transfer to adjacent regions [47]. Thus, the delamination of fibres from their matrix is a significant step in the failure process, which has a negative impact on the material’s strength [48]. This is not easy to identify during servicing.

## 5. Modelling of Failure Energy of PAFC

In order to determine the amount of PAFC specimens that absorb the impact energy, two primary components need to be taken into consideration: the energy necessary to fracture the concrete matrix and the energy required to remove the embedded fibres from the fractured cross-sections. According to the available research [49,50], the mechanism of fibre pullout is often divided into three distinct steps; fibres and matrices cooperating, fibres and matrices separating, and fibres and matrices sliding. The interfacial shear strength of fibre/matrix is considered equal to this research’s corresponding shear bond strength. As a result, the total energy the specimen could absorb during the impact testing may be calculated using Equation (2).

(2) $$U=U_{1}V_{m}+F_{1}U_{2}$$

where U is the specimen’s total absorbed energy, $U_{1}$ is the absorbed crack energy by the reference specimens without fibre, $V_{m}$ is the matrix volume fraction, $F_{1}$ is the number of fibres existing in the cracked cross-section, which can be calculated using Equation (3), $U_{2}$ is the amount of energy needed to remove each fibre.

(3) $$F_{1}=\frac{K_{a}V_{f}}{\pi r^{2}}=\frac{4K_{a}V_{f}}{\pi d^{2}}$$

where $K_{a}$ represents the area of the fractured cross-section of the specimens examined, and $V_{f}$ is the volumetric quantity of fibres present in concrete. The radius and diameter of the utilized fibres are denoted by the symbols *r* and *d*, respectively.

In addition, studies concentrating on assessing energy usage while removing the embedded fibres are reported in the literature [51,52,53]. Assumed by Chawla [54], interfacial frictional shear force $\tau_{i}$ pulls a fibre of a diameter *d* to a distance *x*, total force exerted on the fibre surface that opposes the pullout at this point in time is *τ_i_πd* (*k* − *x*), where *k* is the length of the embedded fibres. The distance *dx* determines the amount of work that is accomplished by the force of the fibre being pulled out far enough is *τ_i_πd* (*k* − *x*) *dx*. The following integration formula may calculate the total effort required to pull the fibre across the specified distance *k*.

(4) $$U_{2}=\overset{l/2}{\underset{0}{\int }}\tau_{i}\pi d\;\left(k-x\right)dx=\frac{\tau_{i}\pi dk^{2}}{2}$$

In this case, we will presume that the fibre will not get broken throughout the pulling-out operation; between zero and *l*/2, its pull-out length may be adjusted, where *l* is the fibre length. This way, the average effort of pullout per fibre may be calculated by integrating *dk* [55].

(5) $$U_{1}=W_{fp}=\frac{1}{l/2}\overset{l/2}{\underset{0}{\int }}\frac{\tau_{i}\pi dk^{2}}{2}dk=\frac{\tau_{i}\pi dl^{2}}{24}$$

where *W_fp_* is energy per fibre. So,

(6) $$U_{2}=W_{fp}=\frac{\tau_{i}\pi dl^{2}}{24}$$

Substituting Equation (6) into Equation (2) gives:

(7) $$U=U_{1}V_{m}+\frac{\tau_{i}l_{1}^{2}K_{a}V_{f}}{6d_{1}}$$

It is important to establish the interfacial bond strength $\tau_{i}$ between the composite matrix and steel fibres and compute the total impact energy absorbed by the PAFC using Equation (7). This may be described as friction between the fibre and the matrix. Matrix and fibre stresses are added to determine ultimate midspan flexural stress. Because of this, the interfacial bond strength may be calculated as follows.

(8) $$\sigma =\frac{1}{2}V_{f}\;g\;\tau_{i}\left(\frac{L_{f}}{d_{f}}\right)+\sigma_{m}\left(1-V_{f}\right)$$

where *σ* is the flexural stress of the fibrous specimen and *σ_m_* is the flexural strength of the reference specimen, *g* = 1.5 [56].

**Figure 11:**
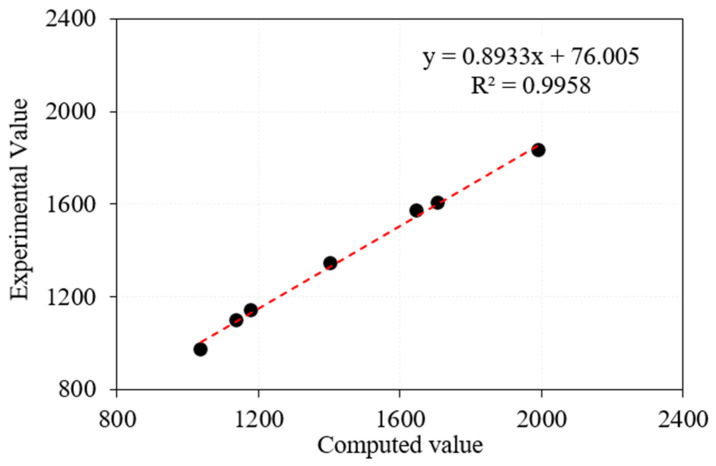
Comparison of impact failure energy (predicted versus experimental).

As illustrated in Figure 11, the theoretical findings from Equation (7) agree with the experimental test findings. The percentage difference between the theoretical and experimental values ranged from 3.5 to 8.11%, indicating reasonable accuracy. It was revealed by Yu et al. [57] that the modelling findings for the smaller specimens were approximately 9.3 percent higher than the experimental results. This discrepancy was ascribed to the energy being lost in the testing instrument. The model has a substantial tendency to underestimate the outcomes of the experiments. This may be the case because the modelling method does not consider the vibration of the testing equipment or the friction between the specimen and the device. Even though the concrete’s impact resistance capacity is fairly high, the drop weight impact instrument may exhibit minor vibrations, which shows that part of the energy is lost in the device.

This model has a few restrictions, including the following: (1) the strength of the interfacial bond may be determined by calculating the flexural strength. As a result, the value of the single loading point’s flexural strength should be utilized to determine the interfacial bonding since impact loading is likewise a single point loading. Flexural strength measured using two loading points does not apply to this model. (2) According to Xu et al. [55], the model findings show a good agreement with the experimental results when the fibre dose is reduced (below 0.6% by volume). The researchers in this study used a greater fibre intake of three percent, which resulted in an 8.11% disparity between their experimental findings and their model results. More research is necessary to determine the appropriate upper limit for the fibre dosage.

## 6. Conclusions

This study investigates the impact behaviour of preplaced aggregate concrete comprising steel fibres and glass fibre mech insertion. Based on the findings obtained from the research, the following conclusions may be inferred.

1. The retained cracking A1 and failure A2 impact numbers were increased as the number of GFM layers was increased. Compared with the F-M0 mixture, the A1 values ranged from a 4 to 8%, and the A2 values from a 9.8 to 35.3%, improvement when the GFM insertion numbers increased from one to three. It is evident that the increasing number of GFM layers exhibited a higher impact strength.
2. Introducing new fibre schemes in PAFC comprising higher fibre dosage in the outer layer and medium in the middle layer positively influenced impact strength compared with the specimen with uniform fibre dosage. For example, the A1 values ranged from 28 to 40% and A2 from 58.8 to 92.2% improvement when the GFM insertion numbers increased from one to three. This is attributed to more fibres in the impact region, which delays the failure by absorbing more energy.
3. Adding GFM and changing fibre dosage in the outer layer of PAFC exhibited a marginal impact in increasing the ductility index compared with the specimen with uniform fibre dosage. The impact ductility index values ranged from 2.15 to 2.56 for the specimens with the same dosage of fibres for all three layers. The specimens with 5, 2 and 5% dosage of fibre from the top, middle and bottom layers, respectively, resulted in ductility index values ranging from 2.53 to 2.80.
4. All non-fibrous specimens failed in a brittle manner, but all fibrous beams failed in a ductile manner. The combinations of GFM and steel fibres dramatically altered the failure mechanism of the material, changing it from a single crack to multiple cracks.
5. Modeling accuracy may be shown in the strong agreement between experimental and computational measurements of failure impact energy. This model has a few drawbacks. The flexural strength may be used to compute the interfacial bond strength. This means that in order to determine interfacial bonding, the single loading point flexural strength value should be employed. This model is not subject to two-point loading flexural strength.
6. Introducing three-layered PAFC comprising higher fibres with a number of GFM additions exhibited a superior impact strength which is essential for many civil engineering applications such as airport runways, industrial flooring and blast walls. Altering fibre dosage, such as by utilizing a higher fibre dosage at the outer layers and a medium fibre dosage at middle layers with the insertion of GFM between the PAFC layers, is this study’s novelty.

## Figures and Tables

**Table 1:** Mixing combinations.

Mixture Id	s/c Ratio	w/c Ratio	Fibre Dosage (%)			GFM	SP
			Top Layer	Middle Layer	Bottom Layer		
F0	1	0.42	0	0	0	0 Layer	0.3
F-M0			4	4	4	0 Layer	0.6
F-M1			4	4	4	1 Layer	0.6
F-M2			4	4	4	2 Layer	0.6
F-M3			4	4	4	3 Layer	0.6
LF-M1			5	2	5	1 Layer	0.6
LF-M2			5	2	5	2 Layer	0.6
LF-M3			5	2	5	3 Layer	0.6

**Table 2:** Impact strength test results.

Mixture Id	A1	A2	U-A1 (J)	U-A2 (J)	IDI	SD (A1)	SD (A2)	COV (A1)	COV (A2)
F0	5	6	102	122	1.20	1.0	1.0	20.0	16.7
F-M0	25	51	509	1038	2.04	2.5	5.0	9.9	9.8
F-M1	26	56	529	1139	2.15	2.0	4.0	7.7	7.3
F-M2	26	58	529	1180	2.23	2.1	4.0	8.1	6.9
F-M3	27	69	549	1404	2.56	1.5	4.0	5.7	5.8
LF-M1	32	81	651	1648	2.53	2.5	5.0	9.9	9.8
LF-M2	33	84	671	1709	2.55	2.0	6.1	6.3	7.6
LF-M3	35	98	712	1994	2.80	2.5	4.6	7.5	5.5

SD: Standard deviation, COV: coefficient of variance.

## Data Availability

Not applicable.
